# Drug Overdose Deaths Before and After Shelter-in-Place Orders During the COVID-19 Pandemic in San Francisco

**DOI:** 10.1001/jamanetworkopen.2021.10452

**Published:** 2021-05-12

**Authors:** Ayesha Appa, Luke N. Rodda, Caroline Cawley, Barry Zevin, Phillip O. Coffin, Monica Gandhi, Elizabeth Imbert

**Affiliations:** 1 Division of HIV, Infectious Diseases, and Global Medicine, University of California San Francisco; 2 Office of the Chief Medical Examiner, San Francisco, California; 3 Department of Laboratory Medicine, University of California, San Francisco; 4 Department of Emergency Medicine, University of California, San Francisco; 5 San Francisco Department of Public Health, San Francisco, California

## Abstract

This cross-sectional study evaluates the association of the shelter-in-place order with fatal drug overdoses in San Francisco, California.

## Introduction

Fatal drug overdoses have been a growing public health crisis for years prior to the COVID-19 pandemic.^1^ In December 2020, the US Centers for Disease Control and Prevention (CDC) issued an advisory that overdose deaths had reached an all-time high, citing increasing synthetic opioid–related deaths.^2^ It is unknown how COVID-19 and health orders to mitigate transmission may be associated with this increase. In this study, we aimed to describe overdose deaths in San Francisco before and after the initial COVID-19 shelter-in-place order to elucidate whether characteristics of fatal overdoses changed during this time to guide future prevention efforts.

## Methods

We performed a cross-sectional study of unintentional fatal overdoses in San Francisco for the 8.5 calendar months before and after the shelter-in-place order on March 17, 2020. Research was conducted in accordance with the Strengthening the Reporting of Observational Studies in Epidemiology (STROBE) reporting guideline. The University of California, San Francisco institutional review board approved decedent research, waiving informed consent; data were not deidentified.

Using data from the Office of the Chief Medical Examiner (OCME), we evaluated unintentional overdose deaths involving fentanyl, heroin, medicinal opioids, methamphetamine, and cocaine. Demographic and toxicological outcomes were identified from forensic findings; cause-of-death determination methods have been previously published.^3,4^ Housing information was collected from OCME investigations and analyzed using a county-level database of social service interactions. We compared overdose death features using χ^2^ tests and calculated normalized death rates using postpandemic overdose deaths and 2019 US Census Bureau data.^5^ To account for temporal variation and illustrate prepandemic trends, we present overdose deaths by month between 2017 and 2020. Statistical significance was set at *P* < .05, and all tests were 2-tailed. Statistical analysis was conducted in Stata version 16.1 (StataCorp).

## Results

In the 8.5 months before and after San Francisco’s COVID-19 shelter-in-place order, 365 and 537 people experienced fatal overdoses, respectively, for a total of 902 deaths. The median (interquartile range) number of weekly overdose deaths was 10 (7-12) before the shelter-in-place order vs 15 (11-18) after the order, representing a 50% increase. Differences in decedent age and sex between time periods were not significant (Table). While the proportion of Black decedents slightly decreased after the shelter-in-place order, the death rate was still disproportionately high (272 per 100 000 Black residents vs 89 per 100 000 White residents). The proportion of decedents experiencing homelessness increased after the shelter-in-place order, from 85 (23%) to 183 (34%) (*P* = .001). The percentage of deaths attributable to fentanyl significantly increased (from 226 [62%] to 388 [72%]; *P* = .001), while the percentage of deaths related to methamphetamine remained unchanged (193 [53%] to 309 [58%]; *P* = .17). The Figure contextualizes deaths by month and year, demonstrating preexisting increases in 2019, with a clear increase in 2020. For example, in May 2019, there were 21 fatal overdoses vs 69 in May 2020.

**Table.** Drug Overdose Death Characteristics in San Francisco, California, in 8.5 Months Before and After COVID-19 Shelter-in-Place Health Order

Characteristic	Unintentional overdose deaths, No. (%)		*P* value^a^
	July 1, 2019, to March 16 2020	March 17 to Novenver 30, 2020	
Total overdose deaths, No.	365	537	<.001
Median overdose deaths per week (IQR)	10 (7-12)	15 (11-18)	<.001
Demographic characteristics			
Age group, y			
<15	0	0	.85
15-24	12 (3)	19 (4)	
25-44	139 (38)	208 (39)	
45-64	171 (47)	257 (48)	
≥65	40 (11)	51 (10)	
Unknown	3 (<1)	2 (<1)	
Sex			
Men	286 (78)	442 (82)	.14
Women	79 (22)	95 (18)	
Race/ethnicity			
Asian	10 (3)	22 (4)	.02
Black	106 (29)	131 (24)	
White/Latinx^b^	212 (58)	353 (66)	
Other	4 (1)	7 (1)	
Unknown	33 (9)	24 (4)	
Decedents experiencing homelessness	85 (23)	183 (34)	.001
Substance implicated as cause of death^c^			
Fentanyl	226 (62)	388 (72)	.001
Heroin	76 (21)	67 (12)	.001
Other opioids	40 (11)	52 (10)	.53
Methamphetamine	193 (53)	309 (58)	.17
Cocaine	184 (50)	185 (34)	<.001
Location of death			
Hospital	41 (11)	79 (15)	.13
Hotel or motel	20 (5)	27 (5)	.77
Outdoors	80 (22)	136 (25)	.24
Shelter	3 (1)	4 (1)	.90
Shelter-in-place sites^d^	NA	21 (4)	NA
Private indoors, eg, residences, commercial buildings	221 (61)	270 (50)	.002

Abbreviations: IQR, interquartile range; NA, not applicable.

^a^ Proportions were compared using χ^2^ tests; medians were compared using Wilcoxon rank sum tests.

^b^ Race/ethnicity data during this time period were not disaggregated to allow for distinction between White and Latinx deaths.

^c^ Not mutually exclusive.

^d^ Shelter-in-place sites were established by the city in April 2020 to place individuals from existing congregate shelters, hospitals, or the street. Individuals susceptible to COVID-19 (ie, those aged >60 years and/or with health conditions) were prioritized.

**Figure.**
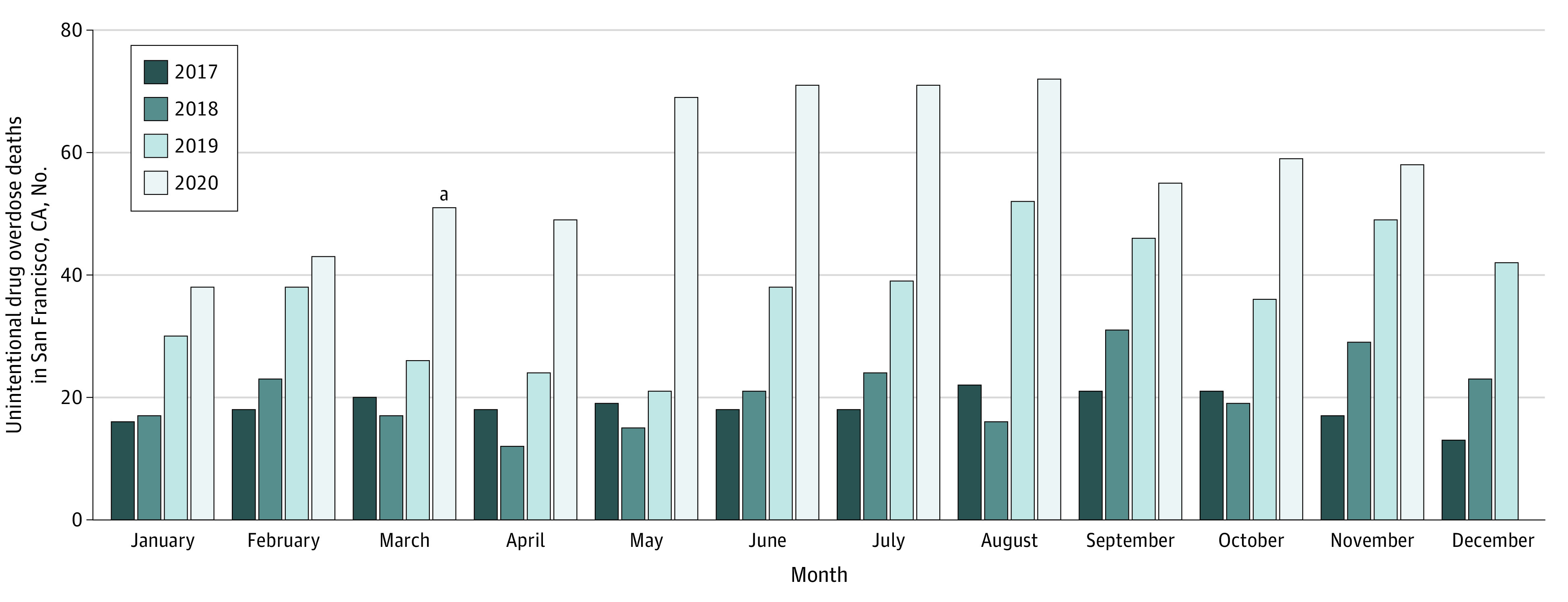
Unintentional Drug Overdose Deaths in San Francisco, California, by Month and Year, January 2017 to November 2020 ^a^The shelter-in-place order went into effect on March 17, 2020.

## Discussion

In this cross-sectional study, we found a continued increase in drug overdose deaths in San Francisco since the onset of COVID-19, with a 50% increase in weekly median overdose deaths. Of note, between March 17 and November 30, 2020, San Francisco recorded 537 drug overdose deaths, while recording 169 deaths due to COVID-19 in the same time period.^6^ The number of fatal overdoses among people experiencing homelessness during COVID-19 doubled.

This follows the increasing trend in 2019 overdose deaths because of fentanyl, which is also associated with the increase in 2020. However, societal disruption related to COVID-19 is likely contributing, as it disproportionately affects people experiencing poverty and marginal housing. Likewise, overdose deaths among Black individuals in San Francisco have been persistently and disproportionally high. Preventing fentanyl-related deaths is of paramount importance, as deaths continue to increase amid the COVID-19 pandemic^2^; however, an ongoing focus on methamphetamine in our region is also needed.

This study was constrained to 1 geographic region, used OCME data (limiting covariate adjustment), and was cross-sectional, precluding causative statements. San Francisco has had a low COVID-19 mortality rate compared with other municipalities and adopted a comprehensive response to the pandemic. Our findings suggest that to complement a strong public health response to COVID-19, there must be more robust overdose prevention for people who use drugs, particularly for people experiencing homelessness, people who identify as Black, and people who use fentanyl and/or stimulants.
